# Causes of Fever in Rural Southern Laos

**DOI:** 10.4269/ajtmh.14-0772

**Published:** 2015-09-02

**Authors:** Mayfong Mayxay, Onanong Sengvilaipaseuth, Anisone Chanthongthip, Audrey Dubot-Pérès, Jean-Marc Rolain, Philippe Parola, Scott B. Craig, Suhella Tulsiani, Mary-Anne Burns, Maniphone Khanthavong, Siamphay Keola, Tiengkham Pongvongsa, Didier Raoult, Sabine Dittrich, Paul N. Newton

**Affiliations:** Microbiology Laboratory, Lao-Oxford-Mahosot Hospital-Wellcome Trust Research Unit, Mahosot Hospital, Vientiane, Lao People's Democratic Republic; Faculty of Postgraduate Studies, University of Health Sciences, Vientiane, Lao People's Democratic Republic; Centre for Tropical Medicine and Global Health, Churchill Hospital, University of Oxford, Oxford, United Kingdom; UMR_D190 “Emergence des Pathologies Virales,” Aix-Marseille University, IRD French Institute of Research for Development, EHESP French School of Public Health, Marseille, France; WHO Collaborating Center for Rickettsioses and other Arthropod-Borne Bacterial Diseases, Aix-Marseille University, Marseille, France; WHO Collaborating Centre for Reference and Research on Leptospirosis, Queensland, Australia; Faculty of Science Health, Education and Engineering, University of the Sunshine Coast, Queensland, Australia; Department of International Health, Immunology and Microbiology, Copenhagen Centre for Disaster Research, University of Copenhagen, Copenhagen, Denmark; Centre of Malariology, Parasitology and Entomology, Vientiane, Lao People's Democratic Republic; Phalanxay District Hospital, Savannakhet Province, Lao People's Democratic Republic; Savannakhet Provincial Malaria Station, Savannakhet Province, Lao People's Democratic Republic

## Abstract

The etiology of fever in rural Lao People's Democratic Republic (Laos) has remained obscure until recently owing to the lack of laboratory facilities. We conducted a study to determine the causes of fever among 229 patients without malaria in Savannakhet Province, southern Laos; 52% had evidence of at least one diagnosis (45% with single and 7% with apparent multiple infections). Among patients with only one diagnosis, dengue (30.1%) was the most common, followed by leptospirosis (7.0%), Japanese encephalitis virus infection (3.5%), scrub typhus (2.6%), spotted fever group infection (0.9%), unspecified flavivirus infection (0.9%), and murine typhus (0.4%). We discuss the empirical treatment of fever in relation to these findings.

With significant reductions in malaria incidence in southeast Asia, the public health importance of understanding the epidemiology of other febrile illnesses has increased. Fever is a common cause of hospital consultation and admission in both adult and pediatric patients in rural Lao People's Democratic Republic (Laos), but its etiology has remained obscure until recently owing to the lack of laboratory diagnostic facilities.^1^ Information on the causes of fever in patients without malaria is urgently needed to guide optimal empirical treatment and for disease surveillance. Among patients admitted to a central hospital in Vientiane, the capital of Laos, *Salmonella enterica* serovar Typhi was the cause of 52% of detected community-acquired bacteremias.^2^ For blood culture-negative and malaria-negative admitted adults, scrub typhus (15%), murine typhus (10%), dengue (10%), leptospirosis (10%), spotted fever group (SFG) infections (3%),^3^ and Japanese encephalitis virus (JEV) infection (2%) (Dubot-Pérès and others, unpublished data) were important causes of fever. A recent prospective study of the causes of non-malarial fever in two provincial hospitals (Luang Namtha in northwest and Salavan in southern Laos) showed that among 41% patients with diagnoses and with exclusion of influenza, the top five diagnoses, when only one etiological agent per patient was identified, were dengue (8%), scrub typhus (7%), JEV (6%), leptospirosis (6%), and bacteremia (2%).^1^ Despite being a small country, the causes of fever between the northerly and southerly sites significantly differed, with JEV infection, typhoid, and leptospirosis more common at Luang Namtha site and dengue and malaria more common at Salavan site.^1^ Here, we report the causes of non-malarial fever in a different rural southern Lao province.

The study was conducted between May–August 2003 and May–August 2004 at Phalanxay District Hospital (∼10 beds), Savannakhet Province, southern Laos (∼605 km southeast of Vientiane, 16.32° N, 106.01° E, and 185 m above sea level) during malaria clinical trials.^4^ Ethical approval was granted by the Faculty of Medical Sciences Ethical Committee, National University of Laos. Patients who had fever (axillary temperature > 37.5°C) at presentation or history of fever for < 21 days with negative malaria blood smears (malaria rapid diagnostic tests [RDTs] were not available in Laos in 2003–2004) and without obvious causes of fever were included provided they or their guardians (in case of children) gave written informed consent. Admission symptoms and signs were recorded on case record forms by two study doctors (Mayfong Mayxay and Maniphone Khanthavong). Venous blood samples at presentation (5 mL for children and 10 mL for adults) and additional 3–5 mL convalescent-phase venous blood samples (collected ∼2 weeks later) were taken and sera were stored at −20°C in the field for 1 month and then at −80°C until analysis. Sera were tested for dengue, JEV, leptospirosis, scrub typhus, murine typhus, and SFG infection. Dengue and JEV enzyme-linked immunosorbent assay (ELISA) kits (Panbio Inc., Brisbane, Australia now Alere Inc., Waltham, MA) were used to investigate dengue and JEV infection.^1^ The Japanese encephalitis-Dengue IgM Combo (E-JED01C) was used to detect and distinguish IgM against dengue and JEV, the Dengue IgG Capture (E-DEN02G) was used to detect high-level anti-dengue IgG (HL-IgG ELISA) in acute secondary dengue infection, the Dengue IgG indirect ELISA (E-DEN01G) was used to detect low-level anti-dengue IgG including IgG from past exposure (LL-IgG ELISA), and the Dengue Early ELISA (E-DEN01P) was used to detect dengue nonstructural protein-1 (NS1) that has high specificity during the first ∼5 days of illness. Interpretative criteria are given in the work of Mayxay and others.^1^ Dengue genome detection and serotyping were performed using TaqMan reverse transcription polymerase chain reaction (RT-PCR) systems as described previously.^5^ Leptospirosis was diagnosed using microscopic agglutination tests (MATs) performed at the WHO Collaborating Center for Reference and Research on Leptospirosis, Queensland Health Forensic and Scientific Services, Queensland, Australia, using the serovars as stated in the work of Syhavong and others.^6^ Leptospiral MATs were regarded as positive if admission serum showed a titer of ≥ 1:400, or if paired sera demonstrated a 4-fold rise. Specific microimunofluorescence assays (IFA) for detection of scrub typhus, murine typhus, and SFG infection were performed in Marseille, France, using whole-cell antigens of *Orientia tsutsugamushi* serotypes Karp, Kato, Gilliam, and Kawasaki and with *Bartonella henselae*, *Coxiella burnetii*, *Rickettsia conorii* subsp. *indica*, *R. felis*, *R. heilongjiangensis*, *R. helvetica*, *R. honei*, *R. japonica*, *Rickettsia* “ATI,” *R. slovaca*, and *R. typhi*.^3^ An IFA result was considered positive if any of the following were detected: 1) positive antibody titers > 1:128 for IgG and > 1:64 for IgM, 2) seroconversion, or 3) > 4-fold increase in titers between acute and the convalescent-phase sera. Admission serum alanine transaminase (ALT), aspartate transaminase (AST), and bilirubin were determined in the laboratory of the Faculty of Tropical Medicine, Mahidol University, Bangkok, Thailand. Blood. Cerebrospinal fluid examinations and cultures were not performed because of the lack of accessible laboratory facilities. The patients were treated according to best local practice and details of treatment recorded.

During the 8 months of this study, malaria was confirmed by microscopic examination for 758/3,767 (20%) of all patients presenting with fever.^7^,^8^ A total of 229 patients (49% aged < 15 years) without evidence for malaria were enrolled (Table 1). The reasons for not having recruited those without malaria were that the patients showed obvious causes of fever, they did not give consent, and/or there was a low probability of follow-up for convalescent samples. Of 229 patients, 128 (56%) had fever (axillary temperature ≥ 37.5°C) at presentation, and the median (range) days of fever was 4 (1–20). Of all studied patients, 120/229 (52%) had evidence of at least one diagnosis (104/229 [45%] with single and 16/229 [7%] with apparent multiple infections). Among patients with only one diagnosis, dengue (30.1% [69/229]) was the most common, followed by leptospirosis (7.0% [16/229]), JEV infection (3.5% [8/229]), scrub typhus (2.6% [6/229]), SFG infection (0.9% [2/229]), unspecified flavivirus infection (0.9% [2/229]), and murine typhus (0.4% [1/229]). For 16 patients with apparent multiple infections (Table 2), all were classified as grade II.^9^ Of 81 patients with dengue diagnosed by IgM and/or IgG serology, 33 (41%) were NS1 positive. Of 48 dengue patients (59% of 81) with sufficient sample for dengue PCR, 8 were positive for dengue (five were DENV1 positive and three were DENV2 positive). Only 5/29 (17%) patients with leptospirosis and 2/14 (14%) with rickettsial infection received appropriate treatment (doxycycline). However, none of the patients died and all were afebrile at the time of follow-up. No patients were lost to follow-up and the median (range) interval until follow-up was 14 (4–75) days.

In this study, half of the patients were assigned an etiological diagnosis, and the most common cause identified was dengue, similar to recent evidence from Salavan (343 km distant from this study site) where 45% of febrile patients had a diagnosis and about one-fifth of them had dengue.^1^ Therefore, dengue is probably one of the most important causes of non-malarial fever in the southern Laos, particularly during the rainy season. Because of the lack of onsite dengue diagnostic tests during this study, only 20% of all 81 confirmed dengue patients (data not shown) were clinically diagnosed as suspected dengue. With new, sensitive and specific RDTs for dengue NS1 detection,^1^,^10^ it is likely that they and fluid management training would be the key clinical interventions for such rural hospitals. Since 2007 all four dengue serotypes have been recorded in Laos,^11^ with dengue 1 being the predominant serotype. In this study, serotypes 1 and 2 were both detected in 2003 and 2004. Dengue has been regarded an urban disease in Laos but these data and data from rural Sayabury Province (northwest Laos) suggest that dengue is also an important rural disease.^12^

As suggested by Mayxay and others,^1^ this study confirms that leptospirosis and rickettsial diseases are important and treatable causes of non-malarial fever in rural Laos and therefore should be considered for patients presenting with acute malaria-negative fever. Given that rapid and accurate malaria and dengue RDTs are now available in Laos, these tests could be performed for acutely febrile patients presenting to hospitals of rural Laos, with dengue RDTs performed if malaria RDTs are negative. If both RDTs are negative, empirical treatment with oral doxycycline would be an appropriate clinical intervention. Cost-benefit analysis is needed to examine the economic and policy implications of such a strategy. This study also suggests that JEV infection is an important cause of acute non-malarial fever in rural southern Laos and that vaccination is likely to reduce JEV undifferentiated fever incidence. With evidence that JEV infection is more common in the north than in the south,^1^ vaccination has been introduced into the six northern provinces of Laos since April 2013. However, with evidence of JEV infection in both Phalanxay and Salavan, it is likely that the infection is widespread in Laos and that vaccination should cover the whole country.

Important limitations of this study include that 1) patients were recruited a decade ago, and the results might not reflect the current situation; 2) patients were enrolled only during the rainy season, and the causes of fever might not reflect those in a complete year cycle; 3) some potential infections and syndromes (influenza, sepsis, central nervous system infection, urinary tract infection, hepatitis, HIV, and tuberculosis) were not investigated because of resource constraints; 4) diagnoses were predominantly based on antibody tests (except dengue NS1 antigen detection—in some instances, antibody detection may be indicative of previous, rather than current, infections but these were overcome where possible by analysis of paired sera and by employing conservative titer values); and 5) the small sample size with non-probability sampling limited the external validity of the results.

This study provides further evidence, from another site in rural Laos, of the importance of dengue, JEV, leptospirosis, and scrub typhus as causes of fever. This is especially important as the former viruses are potentially preventable and the later bacteria treatable with relatively inexpensive, accessible antibiotics. Parallel consensus studies on the etiology of fever at a large number of sites across mainland southeast Asia would lead way to a much needed understanding of the geographical ecology of these pathogens^13^ and how this may influence the empirical treatment policies.^14^

## Figures and Tables

**Table 1:** Demography, admission clinical features, and investigations of the patients (data are shown as number [%] unless otherwise indicated)

Variable	Value
Sex: male/female	129 (57)/99 (43)
Age, median (range): years	15 (1–80)
No. of patients aged < 15 years	113 (49)
Temperature, mean (95% CI): °C	37.7 (37.5–37.8)
Pulse, mean (95% CI): beats per minute	92.8 (90.9–94.7)
Respiratory rate, mean (95% CI): per minute	25.9 (25.1–26.8)
Systolic blood pressure, mean (95% CI): mmHg	104.3 (102.7–106.1)
Diastolic blood pressure, mean (95% CI): mmHg	68.9 (67.5–70.4)
Days of fever, median (range)	4 (1–20)
Glasgow Coma Score: median (range)	15 (7–15)
Chill	96/227 (42)
Headache*	175/222 (79)
Dizziness*	142/222 (64)
Weakness	181/227 (80)
Nausea*	93/221 (42)
Vomiting	82/227 (36)
Abdominal pain*	63/222 (28)
Diarrhea	44/227 (19)
Anorexia	135/227 (59)
Cough	88/227 (39)
Sore throat	66/222 (30)
Runny nose	51/227 (39)
Difficult breathing	39/227 (17)
Back pain	74/222 (33)
Myalgia	97/222 (44)
Arthralgia	87/222 (39)
Dysuria	19/222 (9)
Convulsion	3/227 (1)
Drowsiness	4/227 (2)
Abnormal lungs	3/225 (1)
Abdominal tenderness	7/224 (3)
Hepatomegaly	8/228 (3.5)
Splenomegaly	10/228 (4)
Lymphadenopathy	9/225 (4)
Rash	7/225 (3)
Tourniquet test + ve	23/224 (10)
Haematocrit, mean (95% CI): %	37.8 (37.1–38.5)
AST, median (range): IU/L	25 (7–576)
ALT, median (range): IU/L	7 (1–130)
Total bilirubin, median (range): mg/dL	0.2 (0–8.8)
Direct bilirubin, median (range): mg/dL	0 (0–6.8)

ALT = alanine transaminase; AST = aspartate transaminase; CI = confidence interval.

* Only those aged > 4 years were asked about these symptoms.

**Table 2:** Details of apparent multiple infections among 16 patients (data are shown as number [%])

Variable	Value
Dengue + leptospirosis	10/16 (62.5)
Murine typhus + SFG infection	3/16 (19)
Dengue + scrub typhus	1/16 (6)
Leptospirosis + flavivirus infection	1/16 (6)
Dengue + leptospirosis + murine typhus	1/16 (6)

SFG = spotted fever group.

All 16 patients were classed as grade II.^9^
